# Using MRI to Study High Pressure Assisted Nutrient Infusion

**DOI:** 10.3390/molecules27227972

**Published:** 2022-11-17

**Authors:** Julia D. Kerr, Daniel M. Gruber, Matthew P. Augustine

**Affiliations:** Department of Chemistry, University of California, Davis, CA 95616, USA

**Keywords:** MRI, high pressure, high pressure assisted infusion, diffusion

## Abstract

High pressure assisted infusion of nutrients into food was in situ monitored with magnetic resonance imaging (MRI). Modification of an off-the-shelf pressure reactor with an MRI detection circuit provided a large enough volume to accommodate food. The model food used here was peeled apple flesh as it is considered as a good mimic for fibrous food. The nuclear spin relaxation properties of the water surrounding the apple flesh were enhanced by adding paramagnetic manganese cations. In this way, MRI relaxation contrast can be used to monitor the location of doped bulk water in and around the apple flesh during pressurization. This work tracked the efficiency of pressure induced nutrient infusion in situ, demonstrating that pressure gating and ramping offer no nutrient mass transport advantage over operation at constant pressure and that the presence of a peel expectedly disrupts solute transport into the fruit. High pressure assisted infusion, with all pressurization strategies shown here, yielded nearly 100-fold faster infusion times than at ambient pressure.

## 1. Introduction

Increasing the nutritional value of food is a goal of modern food science [1]. This can be accomplished with natural hybridization/evolution techniques [2] or genetic modifications [3] prior to growth, by controlling the fertilizers/nutrients provided [4] or the environmental conditions [5] during growth, by using chemistry during manufacturing and processing [6], and finally through preparation prior to consumption [7]. This work uses magnetic resonance imaging (MRI) to in situ characterize the high-pressure infusion of nutrients into food matrices. High pressure assisted infusion is a mechanical technique that places small molecules or nutrients such as vitamins or supplements into food, essentially making ‘superfoods’ without altering genetic material. With the distrust of genetically modified organisms (GMOs) in the food sector [8], high pressure assisted infusion presents a pathway to increase the nutritional value of food while maintaining a non-GMO label [9]. This approach has successfully been demonstrated in model fruit-based systems including the infusion of quercetin into cranberries [10], bioactive compounds into solid apples [11], and calcium into carrots [12]. A particularly nice feature of all this work is that the pressurization systems required to accomplish high pressure infusion are already used commercially in the food industry for high pressure food processing [13]. High pressure food processing is a cold pasteurization technique used to sterilize foodstuff such as hummus, guacamole, and baby food [14]. For high pressure infusion, the fruit is typically placed in a bath containing the nutrients in water and the entire fruit/bath complex is housed in a high-pressure food processor [13]. Application of pressure essentially squeezes the nutrient filled solution into the fruit. 

A consequence of using industry standard high-pressure food processors to experimentally understand high pressure infusion is the restricted sample access. Once placed into the pressurization space, the sample cannot be examined until removed. Thus, any and all available information regarding the mass transport mechanisms involving the infusion of nutrients from the bath into the fruit involves destructive cross sectioning studies of the fruit post pressurization or after removal from the high-pressure food processor. Here, separate fruit cross sections are typically homogenized, extracted with some solvents, and explored with gas or liquid chromatography coupled with mass spectrometry [9,10,12]. By calibrating the instruments to detect the infused nutrient, the cross-sectional density of the nutrients can be physically determined. Repeating the pressurization for a longer period of time on similarly sized fruit provides a labor-intensive estimate of pressure enabled nutrient transport. Indeed, this approach was used to estimate the pressure induced diffusion of quercetin in cranberries by Nitin et al. [10].

Approaches currently exist and have been reported for the non-destructive, in situ monitoring of water uptake in dry beans by MRI, a very similar system to the one investigated here [15]. That study was limited to the infusion of plain water at ambient conditions. Other strategies have also been reported for exploring high pressure processes using MRI [16,17]. However, these approaches have not been extended to study nutrient infusion into food products, likely due to the fact that the pressure cells were custom-built to pursue other problems. 

The purpose of the work reported here is to demonstrate that the combination of a high volume, off-the- shelf pressure reactor modified to accommodate a nuclear magnetic resonance probe circuit [18], with conventional MRI [19], can be used to study high pressure nutrient infusion into food. The pressure reactor/probe has already been used to study nuclear spin relaxation at pressures commonly used for high pressure food processing and is a natural choice for this work given the large sample volume [18].

## 2. Non-Invasive Infusion Measurements through MRI

Magnetic resonance imaging is a useful non-invasive way to explore the proton density inside an object [20]. This fundamental property is why MRI is so useful in medicine. The proton or water density of bone is smaller than in muscle or blood, thus a spatial estimate of this density provides a pixel-by-pixel picture of human anatomy [21]. The intensity of an MRI image pixel depends primarily on proton density, but in the case where drastically different spin relaxation times are present, it can also depend on these times [22]. This feature of MRI was exploited in this work by completely immersing the apple flesh into Mn^2+^ doped water. Mn^2+^ doped water has drastically decreased relaxation times due to the paramagnetic properties of the Mn^2+^ ion [20]. In this way, the doped water can act as a magnetic resonance contrast reagent. Here, the relaxation time for water in the apple flesh is often in excess of 1.5 s while the ca. 1 mM Mn^2+^ solutions yielded the much shorter ca. 150 ms long value. By performing the MRI pulse sequence with a very short recycle delay, the long lifetime apple flesh-based water signal was eliminated from the image while the shorter lifetime, external, Mn^2+^ doped, bulk water signal was displayed [21,22]. Hence, the apple, or region of interest, appeared as a negative space image.

## 3. Materials and Methods

Crystalline manganese chloride tetrahydrate, MnCl_2_·4H_2_O, from Fisher Scientific (Waltham, MA, USA) and hexane from Sigma Aldrich (St. Louis, MO, USA) were both used as received. Gala apples, the fruit used in this study, were obtained from a local grocery store. Hexane was used as the pressure transmission fluid in a syringe pump pressurization system that created hydrostatic pressure inside the pressure reactor/probe. Details regarding the operation of the pressure reactor/probe are provided elsewhere [18].

**Figure 1:**
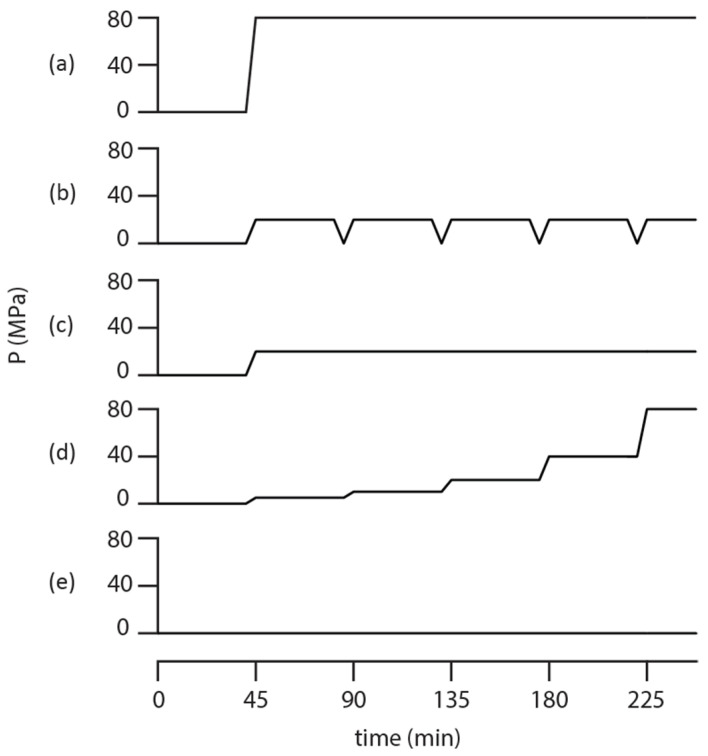
Timing diagram comparing the static 80 MPa pressure in (**a**), cycled 20 MPa pressure in (**b**), static 20 MPa pressure in (**c**), ramped pressure up to 80 MPa in (**d**), and ambient pressure in (**e**) pressurization strategies. Each pressurization run typically lasted 4.5 h while the pressure changes indicated in the diagram, which included a 5 min equilibration period, lasted roughly 10 min.

Cubic, 6–9 mm side length, sections of freshly peeled apple flesh, or apple flesh with peel on one side, were cut and immediately placed into the MRI detection coil inside the pressure reactor/probe. The pressure reactor/probe was then completely filled with 1 mM Mn^2+^ solution in water, closed, attached to the pressure transfer line filled with hexane, and mounted inside a 0.09 T SMIS imaging electromagnet set to operate at a 4 MHz ^1^H Larmor frequency and controlled with a Tecmag (Houston, TX, USA) Redstone MRI spectrometer. Each of the four pressurization profiles shown in Figure 1 as well as the ambient condition study involved recording six images. The four pressurization strategies shown in Figure 1 were undertaken on the model apple system and monitored in situ with MRI in order to determine which pressurization strategy most efficiently infused the solution into the apple flesh. 

The first image was recorded at zero time and the remaining five at 35 min intervals. As the pressure reactor/probe pressurization and depressurization took roughly 2–5 min depending on the size of the pressure differential and the entire assembly was allowed to equilibrate for 5 min following any pressure change, a given pressure run took roughly 4.5 h. All images were obtained using a single gradient-refocused spin echo in the z-direction with a resolution of 0.09 mm and phase encoding in the x- and y-directions, each with a resolution of 0.26 mm. All images were obtained with phase encoding and involved eight signal averages at each point. Longitudinal T_1_ and transverse T_2_ relaxation times were determined in the usual way from the saturation recovery and Carr–Purcell–Meiboom–Gill pulse sequences [20]. All data manipulation and modeling was accomplished with MATLAB™ 2022b (Natick, MA, USA). Regression of the measured normalized signal area included terms up to n = 200 in Equation (2).

## 4. Results and Discussion

**Figure 2:**
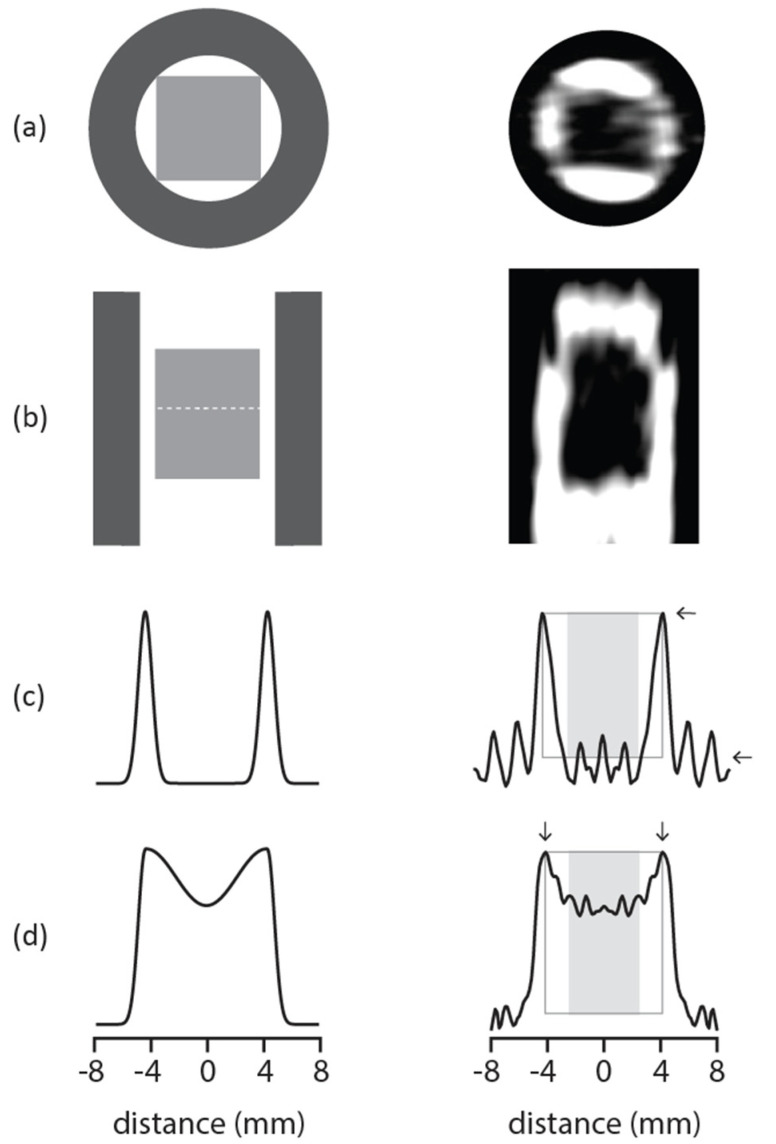
The graphics on the left are illustrative and can be used to understand the real MRI data shown on the right. The real MRI data on the right in (**a**–**c**) were obtained at time 0 prior to pressurization. The data on the right of (**d**) was taken at 80 MPa after 140 min of pressurization. The top and side views of a cubic apple section are described in (**a**,**b**) by the gray solid box on the left and in the corresponding dark squares in the right column images. The white region in these 2D diagrams in both columns corresponds to the bulk doped solution outside the cubic apple section. The 1D slices shown in (**c**,**d**) were obtained at the location of the white dashed line in the left (**b**) image. The gray box bounded by small arrows on the right-hand side of (**c**,**d**) indicates the image region where Equation (1) is valid while the solid gray box defines the range 0 < α < 1 in Equation (2). The arrows are included to specify the box boundary.

The primary goal of this work was to demonstrate that MRI could be used to in situ study the high-pressure infusion of nutrients into food. The model food system used in this work was peeled apple flesh. Since most interesting nutrients are dissolved in water, the problem was simplified to spatially track how bulk water from outside the fruit was transported into the fruit. By adding paramagnetic Mn^2+^ ions to bulk water, the relaxation times shortened to much less than those for water inside the fruit. At the magnetic field strength used here, the 1 mM Mn^2+^ doped water yielded T_1_ = 145 ms and T_2_ = 18 ms while the apple bound water provided T_1_ = T_2_ = 2 s. This fact, along with the relaxation time contrast capability of MRI, enables discrimination and thus the ability to selectively observe the bulk water slowly penetrating the fruit with added pressure. The real MRI data shown on the right-hand side of Figure 2 demonstrated that MRI can detect the fruit, as a low intensity square hole, from both the top and side views in (a) and (b), respectively. 

It stands to reason that these dark square holes of low intensity will become lighter over days at ambient pressure, while osmosis occurs, or over hours at elevated pressure, through high pressure assisted infusion due to the transport of the doped water into the apple flesh. The left-hand column in Figure 2 graphically describes this process. 

The cubic apple flesh inside the cylindrical pressure reactor/probe is shown as the light gray square in the top and side views in Figure 2a and Figure 2b, respectively. The dark gray in this image represents the MRI detection coil. The right-hand column in Figure 2 shows real MRI data. The white space corresponds to the Mn^2+^ doped water that at time zero prior to pressurization is completely outside the fruit. A one-dimensional (1D) slice through the two-dimensional (2D) image at the position of the dashed white line in Figure 2b yields Figure 2c at time zero and Figure 2d at some time after pressurization. Figure 2c,d in the left-hand column show illustrations of the expected 1D slices before and after pressure infusion, while the same rows in the right-hand column show the data obtained before and after 80 MPa of pressure was applied to a cube of apple flesh in Mn^2+^ doped water. The slices at zero time and 140 min of the pressurization cycle on the right in Figure 2c and Figure 2d, respectively, were chosen to reinforce the graphic on the left. The figure provided in the Supplementary Materials shows all of the one-dimensional image slices for the pressurization cycles used in this work.

It is the change in the 1D slice shape as a function of time during pressurization in Figure 2c,d that provides information regarding nutrient infusion. Prior to pressurization, in Figure 2c, the bulk Mn^2+^ doped water was outside the fruit. Increasing the pressure and waiting led to the 1D slice in Figure 2d suggesting that the added pressure enabled the bulk, short relaxation time, Mn^2+^ doped water to infuse into the apple flesh.

**Figure 3:**
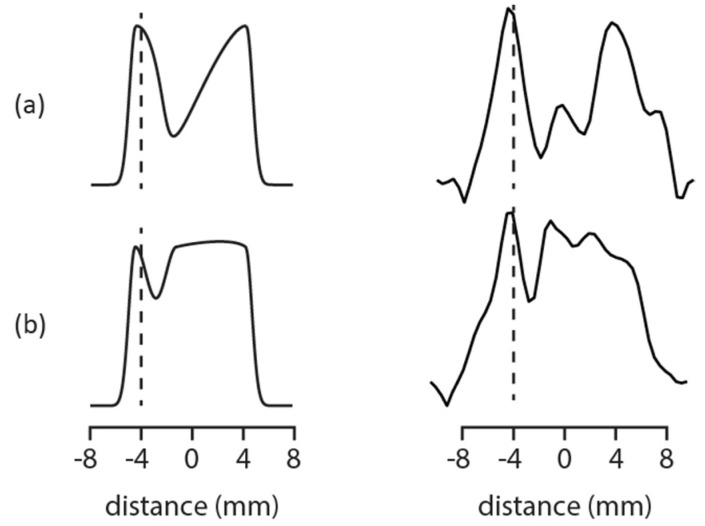
The illustrative 1D slices on the left can be used to understand the real MRI data on the right obtained at 80 MPa applied external pressure. The peel is located at −4 mm, denoted by the dotted line, in all images. The 1D image at 40 min into the pressure run in (**a**) appears to fill in from the side opposite the peel in (**b**) obtained after 160 min at pressure.

The importance of tracking the infusion profiles into fruit flesh can be seen when a less permeable membrane is added to the system, like a peel. This can be seen in Figure 3. The illustrations on the left-hand side of Figure 3 can be used to better understand the real MRI data on the right-hand side of Figure 3. Both columns, illustrative (left column) and real MRI data (right column), describe what happens in the case of when permeability is non-uniform across a food matrix during high pressure assisted infusion. Figure 3 shows 1D infusion profiles at 40 min and 160 min at 80 MPa applied pressure. 

At time zero, the bulk Mn^2+^ doped water was isolated outside the apple section. At later times, some Mn^2+^ doped water infused from all sides except through the peel, thus there was a buildup of signal on the right-hand side of the image. The actual MRI data shown on the right-hand side of Figure 3 are consistent with this prediction, suggesting that the apple peel was less permeable to infusion of the bulk Mn^2+^ doped water. This observation was consistent with infusion studies in cranberry where either the skin needed to be removed or disrupted with sandpaper to increase the nutrient permeability [2,10].

It is the analyses of 1D slices such as those shown in the right column of Figure 2c,d obtained as a function of time during the pressurization cycles shown in Figure 1 that are critical for determining how to efficiently infuse nutrients into the fruit flesh. At time zero in Figure 2c there was no presence of doped water inside the fruit while after 140 min in Figure 2d, the amount of signal in the region of the shaded box increased. At time zero, before pressurization, the amount of signal in the area indicated by the box was zero, at intermediate times non-zero, and at times near the end of the 4.5 h experiment time, one, if the gray box height (and 1D slice maximum) and width were both normalized to one.

The use of MRI allows for the critical first step of qualitatively identifying the mechanism of infusion. The MRI results suggest that the infusion occurs symmetrically from the outside, provided that a barrier such as the peel does not impede infusion. However, a useful and simple parameter is also needed in order to provide a direct comparison between the different pressurization schemes. A natural way to determine this parameter is to adapt well-known diffusion models. Among other things, such models have been successfully applied to the exploration of confined diffusion in porous solids—a environment not too dissimilar to this one—as well as space charges in fluids held at high electric potential [23,24,25,26]. The adapted model, reflecting the experimental reality of the pressure reactor and the sample apple section, is now described.

In the experimental limit here, where the amount of doped water inside the fruit at maximum infusion is much less than the doped water inside the entire pressure reactor/probe volume, it is reasonable to assume that the concentration of doped water outside the fruit remains constant. Moreover, in this study, all experiments showed that the fruit size remained constant throughout the experiment. Since the size of the fruit remains constant and the concentration of the doped water remains constant, a model based on edge sourced one-dimensional bounded diffusion is the most natural [27].

(1) $$P\left(x,t\right)=\frac{1}{\ell }\overset{\infty }{\underset{n=0}{\sum }}\cos\left[2\pi n\left(\frac{x}{\ell }+\frac{1}{2}\right)\right]e^{-{\left(2\pi n/\ell \right)}^{2}D_{\inf}t}$$

where the distribution P(x,t) corresponds to the concentration of doped water inside the fruit flesh at position x as a function of the time t; ℓ is the side length of the cubic fruit flesh; and D_inf_ is a pressure dependent infusion coefficient. In this way, P(x,t) describes the MRI signal from the 1D slices in Figure 2c,d in the region of the gray box bounded by small arrows. The distribution in Equation (1) can be used to model the normalized signal obtained by taking the shaded area only. In terms of Equation (1), this signal is

(2) $$\xi \left(t\right)=\frac{\int_{-\alpha \ell /2}^{\alpha \ell /2}P(x,t)\mathrm{dx}}{\alpha \ell P\left(\ell /2,t\right)}=\frac{1+\sum_{n=1}^{\infty }{\left(-1\right)}^{n}\frac{\sin(\alpha n\pi )}{\alpha n\pi }e^{-{\left(2\pi n/\ell \right)}^{2}D_{\inf}\;t}}{1+\sum_{n=1}^{\infty }e^{-{\left(2\pi n/\ell \right)}^{2}D_{\inf}\;t}}$$

where αℓ is the horizontal size of the shaded gray box in Figure 2c,d. A comparison of the normalized area of the 1D image slices obtained during the pressurization schemes shown in Figure 1 to this equation was used to obtain a pressure dependent infusion coefficient D_inf_. This coefficient describes how fast infusion takes place inside the apple flesh. It is a numerical representation of infusion efficiency. D_inf_ is calculated from the 1D line image slices obtained from the 3D MRI images obtained during pressurization. With knowledge of the fruit size ℓ, the saturation time can be defined as T_sat_ = ℓ^2^/(4π^2^D_inf_). T_sat_ describes the time it takes for the apple flesh to become completely saturated from infusion. This can be seen from the analytical definition of T_sat_ given that the time to saturation is directly linked to the pressure dependent infusion coefficient, D_inf_.

The secondary, but equally important, goal of this work was to use the high-pressure MRI experiment to determine which pressurization strategy in Figure 1 most efficiently infused nutrients into food. From the point of view of this experiment, the goal was to find which pressurization strategy in Figure 1 put the most Mn^2+^ doped water into the fruit in the shortest amount of time. Four different pressurization strategies and a study at ambient conditions as shown in Figure 1a–e were explored here. The two different runs with high and intermediate applied pressure in Figure 1a,c provide the pressure dependent infusion details. The literature suggests [10] that nutrient infusion is more readily accomplished by either gating the applied pressure or by slowly ramping the pressure from zero to a final value. A comparison of the MRI results obtained with the strategies in Figure 1b,c and between Figure 1a,d revealed whether or not pressure gating or ramping improved infusion efficiency. A comparison of the four pressurization strategies in Figure 1a–d to ambient pressure, Figure 1e, revealed how any pressurization strategy compared to the infusion with a lack of externally added pressure. As above-mentioned, the various pressurization strategies in Figure 1 were compared by modeling the area under the 1D image slice as a function of time during the pressurization with Equation (2). Other data models could be used, however, this one was chosen for basic simplicity. The primary output parameter is an infusion coefficient, D_inf_, a number that has physical meaning as it is essentially a pressure-driven diffusion coefficient. The results of this modeling effort applied to the pressurization strategies shown in Figure 1 are summarized in Table 1 with 95% confidence limits. 

Here, the scaling factor α relates to the size of the shaded, integrated area shown in the right-hand column in Figure 2c,d. A value of α = 1 considers the entire fruit section while α = 0.5 limits the area to the center half of the fruit section. The size of D_inf_ reports on how fast the Mn^2+^ doped water enters the fruit and, with knowledge of the fruit size, the time it takes to saturate the fruit, T_sat_, can be calculated. According to the results in Table 1, the higher the pressure, the more efficient the infusion. Any pressurization strategy increases the efficiency of infusion over ambient pressure. There was a ca. 90–500-fold decrease in time to saturation when compared to the ambient pressure conditions. Both D_inf_ and T_sat_ were ca. 4-times more efficient for operation at a constant 80 MPa applied pressure in comparison to 20 MPa for the pressure cycles in rows (a) and (c), respectively. Gating the pressure did not seem to further enable mass transport into the fruit, as suggested by the similar D_inf_ and T_sat_ values for a constant versus gated 20 MPa applied pressure in rows (c) and (d) in Table 1, respectively. Ramping the pressure in Figure 1d also did not encourage mass transport as the D_inf_ and T_sat_ values in row (d) in Table 1 were respectively much smaller and larger than for the results for the constant applied 80 MPa pressure in row (a). The data summarized in Table 1 strongly suggest that the most efficient way to infuse solutes into peeled sliced fruit with pressure is with as high a constant pressure as possible. There will of course be a tipping point where the applied pressure is so high that the fruit shape is physically changed (e.g., smashing or crushing). Such physical changes were not observed in any of the studies reported here.

Although the data model in Equation (2) is arguably primitive, it does provide a simple and fair way to compare 1D images obtained through a pressure run in terms of a physically meaningful infusion coefficient D_inf_. It should be clear that D_inf_ on its own is a fitting parameter and not the same as a real diffusion coefficient. The D_inf_ values reported in Table 1 are specific to the case of pressure infusion and by nature are not measured at equilibrium like a true diffusion coefficient. With the exception of the final, ambient condition results, it is interesting that the D_inf_ values are largely consistent with the values for solute diffusivity inside fibrous food [28]. As expected, the diffusivity of solutes in food was larger at higher temperature at equilibrium, consistent with the increased diffusivity of doped water into apple recognized at a higher applied pressure away from equilibrium. The higher applied pressure forces more solute into the food sample in a shorter time period. The anomalously small D_inf_ and long T_sat_ values reported in Table 1 for ambient conditions are likely a function of the experiment time. As above-mentioned, all pressure runs were completed in 4.5 h to afford a fair comparison between the pressurization strategies. As shown in the Supplementary Figure S1, all pressure cycles at least partially filled the images in this 4.5 h time except for ambient conditions, where there was at best a minor change in the measured data. It was this minor change that was captured by the model and reported in Table 1. To obtain a better estimate of the ambient condition infusion performance, a situation not of interest here, a much longer experiment time to the order of days is required. This longer required time to achieve any noticeable nutrient infusion at ambient conditions underscores how efficient and useful the application of any external pressure is to nutrient infusion into food. The values for the scaling factor α are also sensible given the spatial regions explored in the 1D image slices. The α parameter corresponds to the portion of the image used to determine D_inf_ values and pertains to the location of the infusion boundaries and the physical size of the apple region within the field of view. This is why smaller α values were used for smaller apple sections. Analysis of the 1D images for all of the pressure cycles except (d) involved using half of the fruit section. For pressure cycle (d), involving a smaller apple section, a smaller α = 0.23 integration region was used.

The two obvious limitations of this work were the numerical modeling of Mn^2+^ doped water penetration into the fruit captured by Equation (2) and the idea that nutrients and bulk water similarly pass into fruit with added pressure. As above-mentioned, the numerical model of edge-sourced bounded 1D diffusion was chosen for its simplicity and the similarity of the distributions calculated from Equation (1) and the measured data such as those shown in the right column in Figure 2c,d. The model clearly neglects nutrient filling the fruit from directions perpendicular to the 1D MRI slice direction. Accounting for this fact in addition to numerically solving the diffusion equation with proper boundary conditions would clearly help in properly understanding the evolution of the 1D images as a function of time. However, since the end-product will still be an infusion coefficient that can be used to decide which pressurization strategy in Figure 1 is the most efficient, this was not pursued here. The relevance of this work relies on the idea that real nutrients will pass into the fruit with the bulk water and that by tracking how the bulk water infuses into the fruit matrix, the location of the nutrients is inferred. To track the bulk water, paramagnetic Mn^2+^ cations were added to enhance nuclear spin relaxation. These ions, like nutrients, are transported into the fruit flesh with the bulk water at pressure. It is likely that larger nutrients such as soluble proteins will not permeate the fruit flesh in the same way as the smaller Mn^2+^ cations and water. This can, in principle, be explored by repeating this work with a larger, water stable, non-reactive paramagnetic macromolecule or an electron spin labeled nutrient that replaces the Mn^2+^ cation. A comparison of the 1D images at constant pressure and time as a function of paramagnetic solute size would answer this question and is the subject of future work. 

Finally, having validated the nature of infusion with MRI, additional future work could involve developing a simpler time domain NMR-based method for rapidly monitoring nutrient infusion. The results of these measurements could then be validated with the simple one-dimensional diffusion model. However, the time and cost saving advantages of using time domain NMR to measure diffusion coefficients will be offset by the formidable challenges associated with eddy current generation resulting from strong, rapid, pulsed gradients in a conductive pressure reactor. Issues with dynamic range also make the application of standard relaxometry difficult because the weak signal of interest is overwhelmed by the free water background. Since standard time domain experiments are designed to explore samples at equilibrium and the process of nutrient infusion here is far from equilibrium, the development of additional methods would have to be accomplished to make time domain NMR feasible in this application.

## 5. Conclusions

This work demonstrates that MRI combined with an off-the-shelf pressure reactor customized with an MRI detection circuit [18] can be used to study the high-pressure infusion of nutrients into food. It was also determined that any pressure strategy outperformed ambient pressure infusion, but that neither pressure gating nor ramping offered any obvious advantage among the high-pressure strategies for nutrient infusion performance. Moreover, operation at the highest pressure possible should provide the best infusion performance as long as the fruit is not physically changed or crushed. Consistent with the literature [10,11], the MRI results for unpeeled fruit suggests that the peel significantly attenuates the infusion of bulk Mn^2+^ doped water into the apple section. Future work will consider the effect of peel treatment on mass transport. With this MRI approach, entire small fruits such as blueberries or cranberries can be placed into the pressure reactor/probe and Mn^2+^ doped water penetration could be tracked as a function of peel treatment, roughness, or perforation. 

## Figures and Tables

**Table 1:** Summary of apple section nutrient infusion parameters.

Pressure Cycle ^†^	ℓ (mm)	α	T_sat_ (h)	D_inf_ (cm^2^/s)
(a), 80 MPa static	8.3	0.52	1.3 ± 0.1	(3.7 ± 0.3) × 10^−6^
(b), 20 MPa gated	8.3	0.52	7.3 ± 0.4	(6.7 ± 0.4) × 10^−7^
(c), 20MPa static	8.9	0.48	6.0 ± 0.4	(9.3 ± 0.7) × 10^−7^
(d), 80 MPa ramped	6.0	0.23	8.4 ± 0.6	(3.0 ± 0.2) × 10^−7^
(e), ambient	7.4	0.48	775 ± 36	(5.0 ± 0.2) × 10^−9^

^†^ See Figure 1 for pressure cycle timing.

## Data Availability

Not applicable.

## Supplementary Materials

The following are available online at https://www.mdpi.com/article/10.3390/molecules27227972/s1, Figure S1: One dimensional image slices during pressurization.

## Sample Availability

Samples of the compounds are not available from the authors.
