# Supplementary material for: Using MRI to Study High Pressure Assisted Nutrient Infusion
Source: Molecules. 2022 Nov 17;27(22):7972. doi: 10.3390/molecules27227972 (PMC9696878; doi:10.3390/molecules27227972)
Supplement: Supplementary file 1 [file molecules-27-07972-s001.zip › molecules-1946131-supplementary.pdf]

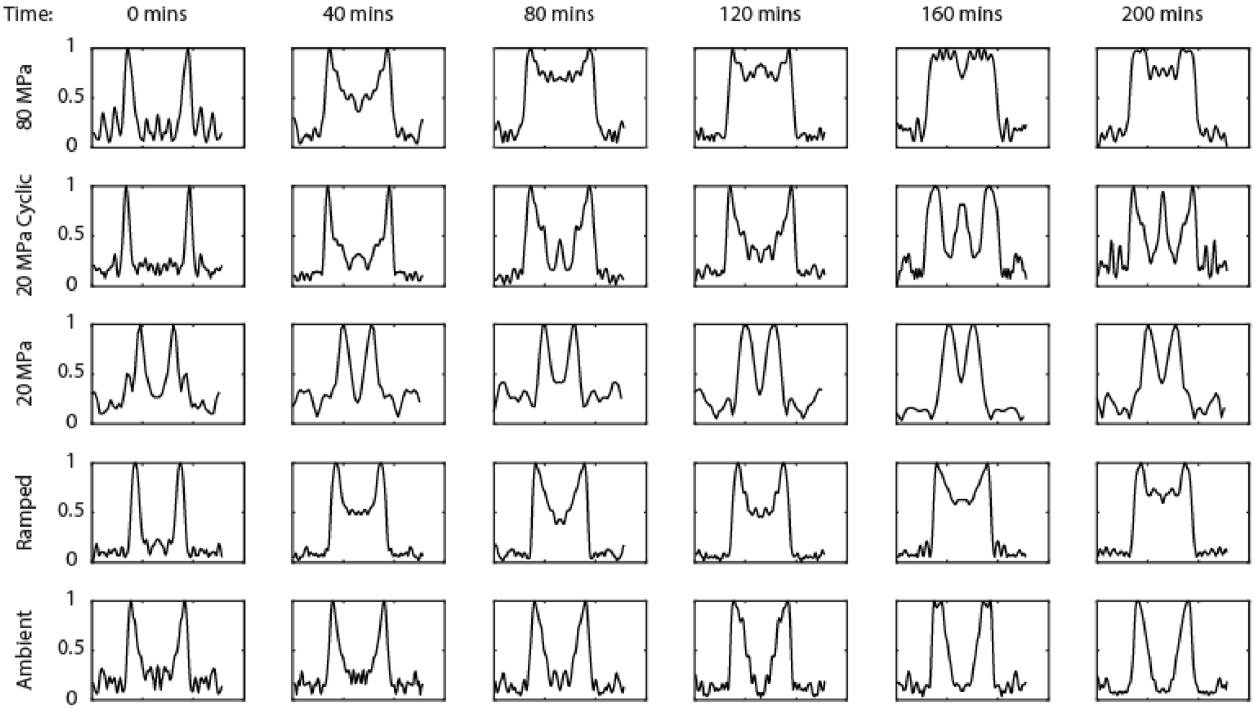

Figure S1. One dimensional magnetic resonance image slices through the center of the apple section at various times during the pressurization cycle. The specific pressurization cycle indicated on the left and the timing at the top is described in the same order in Figure 1. The one dimensional infusion model reduces the pressure cycle data into one parameter D_{inf} for each pressure treatment to enable straightforward comparison.
